# Pomological Characteristics and Ploidy Levels of Japanese Plum (*Prunus salicina* Lindl.) Cultivars Preserved in Poland

**DOI:** 10.3390/plants10050884

**Published:** 2021-04-27

**Authors:** Agnieszka Głowacka, Mirosław Sitarek, Elżbieta Rozpara, Małgorzata Podwyszyńska

**Affiliations:** The National Institute of Horticultural Research, ul. Konstytucji 3 Maja 1/3, 96-100 Skierniewice, Poland; agnieszka.glowacka@inhort.pl (A.G.); elzbieta.rozpara@inhort.pl (E.R.); malgorzata.podwyszynska@inhort.pl (M.P.)

**Keywords:** *Prunus salicina* Lindl., gene bank, cultivars, yield, fruit quality, sharka, ploidy level

## Abstract

Research on the resistance to frost, susceptibility to sharka, flowering biology, fruit setting, yield, and ploidy levels of 36 Japanese plum cultivars (mostly hybrids of *Prunus salicina* with *Prunus cerasifera*) were carried out in 2015–2020 at the Experimental Orchard located in Dąbrowice near Skierniewice. Relatively mild winters with sporadic temperature drops to nearly −21 °C in January of 2017 and 2018 caused slight damage to several cultivars of Japanese plum insufficiently resistant to frost. The trees of most cultivars remained healthy, with no signs of damage. ‘Barkhatnaya’ and ‘Tatyana’ cultivars turned out to be very susceptible to sharka. ‘Herkules’ trees were the most vigorous. ‘Barkhatnaja’, ‘Blue Gigant’, ‘Shater’, and ‘Tatyana’ trees were characterized by weak growth. The trees of Japanese plum started flowering early, usually in the first or second decade of April. Most of the cultivars belonged to early season cultivars, the fruits of which ripened in July. Based on the assessment of tree productivity, ‘Barkhatnaya’, ‘Inese’, ‘Shater’, ‘Tatyana’, and ‘Vanier’ are the best for growing in the climate of Central Europe. ‘Tsernushka’, ‘Chuk’, ‘Dofi Sandra’, ‘Early Golden’, ‘Ewierch Rannyj’, ‘Yevraziya’, ‘Gek’, ‘General’, ‘Kometa’, ‘Kometa Late’, ‘Maschenka’, and ‘Naidyona’ trees also yielded well. ‘Blue Gigant’, ‘Black Amber’, and ‘Herkules’ had the largest fruits, and ‘Chuk’ and ‘Inese’ cultivars produced the smallest fruits. Among the assessed Japanese plum cultivars, those with round fruit, dark skin with various shades of purple, yellow flesh, and A cytometric analysis showed that almost all cultivars are diploid, except for ‘Herkules’ (possibly pentaploid) and ‘Yevraziya’ (possibly hexaploid or aneuploid).

## 1. Introduction

The genus *Prunus*, which belongs to the Rosaceae family, includes over 35 species that are native to Europe, Asia, and America [1,2,3]. The basic chromosome number in *Prunus* is x = 8 [4]. The somatic chromosome number of various *Prunus* species varies from diploid to hexaploid [4,5,6,7]. Most of the species are diploid (2n = 2x = 16), e.g., *P. armeniaca*, *P. avium*, *P. canescens*, *P. cerasifera*, *P. mahaleb*, *P. persica*, *P. spinosa*, and *P. tomentosa.* There are also several tetraploids (2n = 4x = 32), such as *P. cerasus*, *P. fruticosa*, and *P. maackii*, and hexaploids (2n = 6x = 48), such as *P. domestica* and *P. domestica* var. *insititia*. Although *P. salicina* is generally considered to be diploid [5,6], tetraploids have also been recorded in this species [4].

Two types of plum dominate in the world of commercial cultivation. One type is European plums (*Prunus domestica* L.) and the second is Japanese plums (*Prunus salicina* Lindl.) [2,3]. Almost all of the plum cultivars grown in Poland in commercial orchards belong to *P. domestica*. and their cultivation has a long tradition [8,9]. European plum trees start bearing fruit early, and most cultivars yield abundantly and annually. 

Japanese plums (*P. salicina*) are characterized by their high abundance and variability when compared to other tree crops. Japanese plum trees differ from European plum trees in terms of many morphological features, and the fruits are distinguished by size, aroma, color of the skin, and fruit storage capacity [1,3]. Japanese plum cultivars spread to cultivation in the USA thanks to a breeding program initiated in 1875 by Luther Burbank [3,10]. Later, cultivars resulted from crosses between *P. salicina* and American plum (*Prunus americana* Marsh.). They were characterized by increased frost resistance and became common in production. The Japanese plum reached Europe later than the USA and it spread mainly in the Mediterranean countries. In countries of Central and Northeast Europe, interest in this plum was lower due to less favorable climatic conditions. However, in recent decades, the situation has changed due to the gradual warming of the climate and progress in breeding of new cultivars with very attractive fruit and relatively late flowering of trees, which are less exposed to spring frosts. Most of these cultivars result from hybridization between *P. salicina* and either *P. cerasifera* or American plum cultivars. The first Japanese plum trees were brought for testing to The National Institute of Horticultural Research in Skierniewice in the 1970s from Hungary. Because of higher thermal requirements than European plum cultivars and, above all, the very early beginning of vegetation, at that time their flowers were freezing, and the trees either did not bear fruit at all or yield was far from what was expected [8]. 

The results of research that was carried out in the plum collection at the Experimental Orchard in Dąbrowice show that, in recent years, many Japanese plum cultivars have yielded similarly to those of the European plum (*P. domestica*). This article presents an assessment of agronomic value and ploidy levels of 36 Japanese plum cultivars in the climatic conditions of central Poland.

## 2. Results and Discussion

### 2.1. Cold Hardiness

In 2015–2020, the winters were relatively mild. The lowest temperatures with occasional drops to nearly minus 21 °C were recorded in 2017 and 2018. They only caused slight damage to trees of several Japanese plum cultivars, insufficient resistant to frost. Trees of most cultivars remained healthy, with no signs of damage (Table 1). In the analyzed period, winter 2019/2020 was exceptionally warm for this latitude. The lowest temperature was recorded in January, but it was only −4.6 °C. Such conditions meant that trees of three cultivars with a short dormancy period, ‘Formosa’, ‘Gek’, and ‘Dofi Sandra’, started flowering at the end of January. This is a very unfavorable phenomenon, because trees are damaged by subsequent cold periods during winter and early spring, as previously reported by Grzyb and Rozpara [8].

### 2.2. Susceptibility to Plum pox Virus (Sharka)

Sharka is one of the most dangerous plum diseases, which limits the plum cultivation in Europe, including Poland [11,12]. Among the 36 Japanese plum cultivars that were assessed in the experiment, the strongest symptoms of this disease were observed on trees and fruits of ‘Barkhatnaya’ and ‘Tatyana’ cultivars (Table 1). Sharka also appeared on ‘Dofi Sandra’, ‘Early Golden’, ‘Herkules’, and ‘Skoroplodnaya’ trees. Its symptoms were mainly observed on leaves, but, in the case of ‘Early Golden’ and ‘Herkules’ cultivars, they also appeared on fruits (Table 1). In the available literature, there is no information on the susceptibility to sharka of the above-mentioned Japanese plum cultivars.

### 2.3. Tree Vigour

The observations that were carried out in 2015–2020 showed that, in the collection orchard, ‘Herkules’ plum trees had the strongest growth (Table 2). Trees of fifteen out of thirty-six assessed cultivars grew strongly, sixteen cultivars were characterized by moderate growth, and the remaining four-by weak growth. The most of Japanese plum cultivars were dominated by a spreading canopy. ‘Black Amber’, ‘Blue Gigant’, ‘Gauota’, and ‘Herkules’ trees had an upright habit, and ‘Dofi Sandra’, ‘Naidyona’, ‘Skoroplodnaya’, and ‘Vanier’ were conical (Table 2).

### 2.4. Flowering

In the Experimental Orchard at Dąbrowice, the trees of the earliest Japanese plum cultivars began flowering in the first decade of April and ended flowering in the second decade of April (Table 2). Late cultivars started flowering in the second and ended in the third decade of April. In each year of the study, ‘Barkhatnaya’, ‘Tsernushka Rannaya’, ‘Chuk’, and ‘Tatyana’ trees bloomed at the earliest. In contrast, ‘Yevraziya’ trees were the last to bloom. Trees of most cultivars bloomed profusely. When compared to others, only trees of ‘Formosa’ and ‘Herkules’ cultivars bloomed less intensively. The length of flowering period depended on the year and cultivar and, in the years 2015–2020, it was, on average, from eight to 14.3 days. (Table 2). In general, the length of the flowering period of Japanese plum cultivars in Poland was similar to that in Serbia [13,14].

### 2.5. Yield and Ripening Time of Fruit

Trees of twenty-two assessed cultivars started fruiting in the first year after planting, twelve cultivars started fruiting in the second year after planting, and two cultivars, ‘Blue Gigant’ and ‘Yevraziya’, in the third year after planting. Fruit set in individual cultivars in our experiment was varied, because, as a rule, early flowering of trees caused that the buds and flowers were damaged by late spring frost, which was also reported by Butac et al. [15] on the basis of studies carried out in Romania. The ‘Barkhatnaja’, ‘Inese’, ‘Shater’, ‘Tatyana’, and ‘Vanier’ trees were distinguished by regular, very high yields (Table 2). The following trees also yielded satisfactorily: ‘Chuk’, ‘Dofi Sandra’, ‘Early Golden’, ‘Ewierch Rannyj’, ‘Gek’, ‘General’, ‘Kometa’, ‘Kometa Late’, ‘Maschenka’, ‘Naidyona’, ‘Tshernushka’, and ‘Yevraziya’. The yielding results of these cultivars are consistent with the results that were obtained by other authors [13,14,16,17]. The trees of the ‘Formosa’ cultivar had the lowest yields. Moreover, trees of the ‘Black Diamond’, ‘Blue Gigant’, and ‘Herkules’ cultivars yielded poorly and irregularly. In the available literature, there is no information on the yielding of ‘Formosa’, ‘Blue Gigant’, and ‘Herkules’ trees in the conditions of Central and Eastern Europe. Brooks and Olmo [18] report on the high productivity of ‘Black Diamond’ trees. However, this cultivar cannot be classified as abundantly yielding in the climatic conditions of Poland and Hungary [19]. 

The ripening date was constant during the evaluation period. The exception was 2018, when the weather conditions in the growing season meant that the fruit reached harvest maturity approximately two weeks earlier than in other years. Very early, already in the first decade of July, the fruit of ‘Ewierch Rannyj’, ‘Gauota’, ‘Oishi Wase’ ‘Tsernushka’, and ‘Tsernushka Rannaya’ cultivars reached maturity. The ‘Angeleno’ and ‘Friar’ cultivars were included in the group of the latest ones (Table 2). In the climatic conditions of central Poland, the fruits of most Japanese plum cultivars matured a few to several days later than in the conditions of Russia and Ukraine [16,17]. Additionally, the harvest of Japanese plum fruits in Poland takes place later when compared to Hungary [19], Iran [20], or Serbia [13,14]. 

### 2.6. Determination of Fruit Quality 

The mean fruit weight of the Japanese plum cultivars varied from year to year and it depended mainly on the weather conditions and yield. Among the thirty-six cultivars assessed, four cultivars were classified as small-fruited (20–25 g), 15 as medium (26–40 g), nine as large (41–55 g), two as large or very large (56–70 g), and six as very large (over 70 g) (Table 3). The ‘Chuk’ and ‘Inese’ cultivars had the smallest fruits. ‘Blue Gigant’, ‘Black Amber’, and ‘Herkules’ trees produced the largest fruit. The fruit of most cultivars reached a size that was similar to that reported in the literature [8,16,17,18,21]. The exception was ‘Early Golden’ cultivar, which in research conducted in Iran by Pirkhezri et al. [20] had small fruit with low tree productivity. In Poland, the fruits of this cultivar were classified as medium to large in size, depending on the year, and the yielding of trees was high. 

The most numerous group among the studied cultivars were those with round fruits (18 out of 36 assessed) (Table 3). The fruits of five cultivars were round, but flattened at the tops, eight were heart-shaped, and the other five were oval. The skin of half of the cultivars tested was dark with various shades of purple, eleven cultivars had red skin, and three-dark blue (Table 3). Three cultivars: ‘Early Golden’, ‘Gek’, and ‘Shiro’ were covered with a yellow skin, rarely found in plums. Among the tested cultivars, the fruit of twenty-five cultivars had a yellow flesh color. The ‘Black Diamond’, ‘Blue Gigant’, and ‘Santa Rosa’ cultivars had red flesh, and the flesh of the other eight cultivars was yellow-red in color (Table 3).

An important quality parameter of plum fruit is Total Soluble Solids content, which largely determines the fruit’s flavor. This is a cultivar feature, but it also depends on the intensity of fruiting of trees and climatic conditions. Vangdal et al. [22] found a negative correlation between the productivity of trees and quality of plum fruit. Our own results confirm this relationship. In some years, the trees of many Japanese plum cultivars yielded abundantly, which had an adverse effect on the size and taste of plums. An example is 2016, when trees yielded abundantly, but the fruit size and Total Soluble Solids content were lower than in the rest of research years. Total Soluble Solids content of 18–20% almost always guarantees a good taste for plums, according to Kemp and Wustenberghs [23]. In Polish growing conditions, the fruits of cultivars tested contained less sugars, i.e., from 9.6% in the case of ‘Skoroplodnaya’ cultivar to 17.8% in the case of ‘Chuk’ cultivar (Table 3). However, the taste is determined not only by the sugar content in fruit, but also by other characteristics, such as acid content and, in particular, by the sugar-acid ratio. That is why ‘Formosa’, ‘Oishi Wase’, and ‘Ozark Premier’ cultivars had delicious fruit every year. Unfortunately, the climatic conditions in Poland are not conducive to such accumulation of Total Soluble Solids in Japanese plum fruit, such as in Romania [15], Serbia [13,14], or Iran [20].

The dessert fruit, in addition to its attractive appearance and delicious taste, good separation of stone from flesh is essential. In our research, stone was the easiest to separate from flesh in fruit of ‘Slivovidnaya’ cultivar (Table 3). However, as many as twenty-four cultivars had seeds that were firmly attached to flesh, regardless of the year of study. The results of our research are partially consistent with those obtained by other authors [16,17,18,21]. Contrary to results obtained in Poland, Pirkhezri et al. [20] report a very good separation of stone from flesh in fruit of ‘Angeleno’ cultivar. These differences may be caused by climatic conditions that are not favorable for ripening of this cultivar in Poland.

### 2.7. Ploidy Level

**Figure 1:**
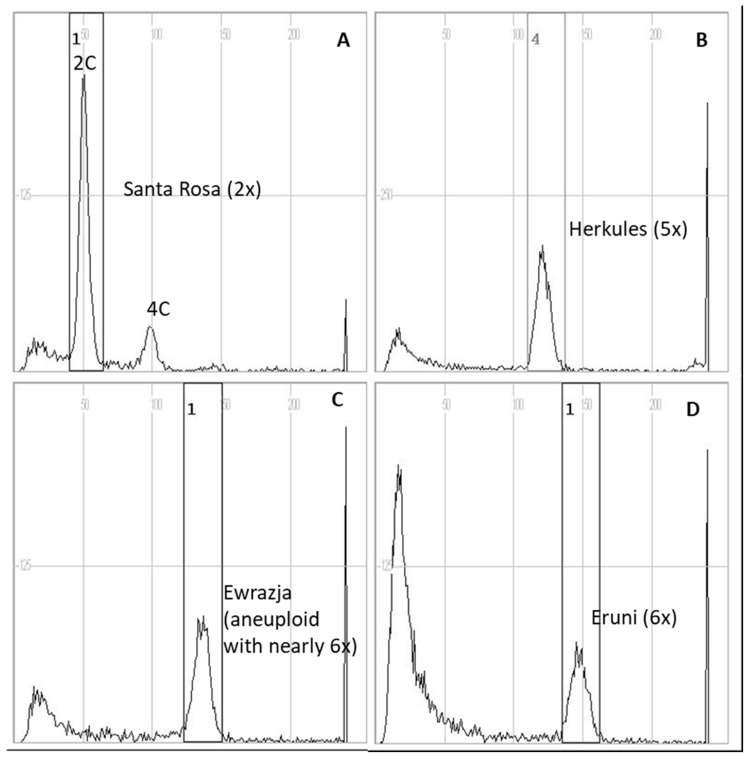
Histograms of ploidy level estimation of Japanese plum cultivars using flow cytometry (FCM): (**A**) external standard diploid cultivar ‘Santa Rosa’; (**B**) pentaploid ‘Herkules’; and, (**C**) aneuploid showing nearly hexaploidy ‘Yevrazija’, and (**D**) reference hexaploid ‘Eruni’.

For the 2C DNA reference diploid cultivar ‘Santa Rosa’, the position of the 2C DNA fluorescence peak of the X axis on the FCM histogram was determined to be 51.3 ± 1.89, and 2C DNA peak for haxaploid ‘Eruni’ was 151.3 ± 2.66 (Figure 1). Among the studied genotypes, 31 genotypes with the peak values on X axis ranging between 50.3 ± 1.66 (recorded for ‘Puteshestvennitsa’) and 54.2 ± 1.78 (for ‘Dofi Sandra’) (on average, 52.1 ± 2.44) were evaluated with high probability as diploids (Table 4). ‘Herkules’ was evaluated to be pentaploid as its peak position was 122.5 ± 1.49 and ‘Yevraziya’ was presumably aneuploid, showing nearly hexaploidy with probably a few chromosomes missing; its peak position was 139.1 ± 2.06. However, the aneuploidy of this cultivar should be confirmed in further studies by the microscopic analysis of the chromosome number. 

Genotypes of higher ploidy levels pentaploid ‘Herkules’ and putative aneuploid hexaploid ‘Yevraziya’ are characterized with the latest date of flowering. This phenomenon of delayed flowering of polyploids within species is often observed [24]. It was reported inter alia for autotetraploids of *Malus* × *domestica* [25], *Gerbera jamesonii* [26], and *Dendranthema nankingense* [27].

It is generally acceptable that the natural polyploid formation within the genus *Prunus* results from both somatic chromosome doubling and union of unreduced gametes with the latter considered to be the most important polyploidisation mechanism [28,29]. The pentaploid ‘Herkules’ is hybrid of hexaploid *P. domestica* ‘Ontario’ and diploid *P. salicina* ‘Formosa’, and its pentaploidy results from pollination with unreduced pollen (2n) of ‘Formosa’ [30,31].

## 3. Materials and Methods

### 3.1. Location and Plant Material 

Research work was conducted in 2015–2020 based on the field experiment that was located at Experimental Orchard in Dąbrowice (central Poland–latitude 145 m, 51°54″ N/20°06″ E). The 36 Japanese plum cultivars were evaluated (Table 5). 

One-year-trees of Japanese plum cultivars (*Prunus salicina* Lindl.), grafted onto ‘Wangenheim Prune’ seedling rootstocks, were planted in the spring of 2013, with a spacing of 4.7 m × 3 m, in a grey-brown podzolic soil. Each cultivar was represented by three freestanding trees. In the first two years after planting, the soil was kept free from weeds by mechanical cultivation. During the following years, soil management included frequent grass mowing in the alleyways and maintenance of 1m wide herbicide strips along the tree rows. The experimental orchard was irrigated with a drip system. Fertility, pest, and disease control were carried out in accordance with the current recommendations for commercial plum orchards and the principles of integrated fruit production.

### 3.2. Weather Conditions

Every year, data on weather conditions were collected through the meteorological station that was located at the Experimental Orchard in Dąbrowice. The collected climatic data were used to assess their impact on health condition and yield of trees, as well as on fruit quality. In the years of research, the average annual rainfall in this area was 446 mm, and the average annual temperature was 9.6 °C. Table 6 presents basic meteorological data for the period 2015–2020.

### 3.3. Tree Growth and Productivity Assessment

In 2015–2020, the following parameters were assessed:

- *Frost damage to trees*—assessed on a 1–9-point rating scale according to Perczak [33].
- *Plum pox virus (sharka) symptoms*—on the basis of inspection performed at the end of June and in the middle of September. The state of trees was determined visually on a 0–3 point rating scale for symptoms on the leaves. The symptoms of the fruit were investigated, when the fruit consumption maturity was reached on a 0–3 point rating scale according to Iliev and Stoev [34].
- *Tree vigor* and *canopy shape* assessed on a 1–9 point rating scale.
- *Flowering time*—was recorded by recommendations of the international working group for pollination: start of flowering–10% open flowers; full bloom–80% of the flower buds on the tree had reached the open flower stage; and, end of flowering–90% of the petal fall [35].

### 3.4. Determination of Fruit Quality

The evaluation of the fruit of each genotype was determined in each year of the study using five samples of 20 randomly picked ripe fruits. The subject of the research was the mean fruit weight and shape of fruit, skin and flesh color, as well as Total Soluble Solids content in fruit (using the ATAGO PR-101 electronic refractometer, Atago Co., Ltd., Tokyo, Japan). 

The evaluation of cultivars in terms of pomological traits was made according to the Plum descriptor that was developed by UPOV (International Union for the Protection of New Varieties Cultivars of Plants).

### 3.5. Ploidy Level

Analysis of ploidy level was performed in 2020 using flow cytometry analysis (FCM). Young leaf samples were randomly collected from two plants of each genotype. Leaf tissue (0.25−0.5 cm^2^) was chopped in a Petri dish in 0.75 mL nuclei isolation Partec buffer (Sysmex Partec GmbH, Münster, Germany) to which 50 μg mL^−1^ 4′,6-diamidino-2-phenylindole (DAPI) and 1% polyvinylpyrrolidone (PVP) were added. After adding 0.75 mL of the isolation buffer, the samples were filtered through a 30 μm filter and then incubated for 45–60 min. in darkness at room temperature. The fluorescence of the nuclei was measured using ploidy analyser CyFlow Ploidy (Sysmex Partec GmbH, Münster, Germany) with UV-LED 365 nm. The data were analyzed by means of software CyView (Sysmex Partec GmbH, Münster, Germany). Samples with at least 2000 nuclei were measured for two leaves of each plant. As external standard of known ploidy levels, diploid cultivar ‘Santa Rosa’ [5] and hexaploid *P. domestica* ‘Eruni’ were used [7].

### 3.6. Statistical Analysis

The obtained results concerning the morphological features of trees are presented numerically in tables. The results of measurements of fruit weight and Total Soluble Solids content were processed using one-way statistical analysis of variance in the Statistica 10 program. The Duncan’s multiple range test was employed at *p* = 0.05 to evaluate the significance of the differences between means.

## 4. Conclusions

The conducted research showed a large diversity of genotypes in the analyzed Japanese plum population. The Japanese plum cultivars differ in terms of tree vigor, frost resistance, susceptibility to sharka, flowering time and intensity, yielding, fruit maturity date, and fruit quality characteristics. Based on the assessment of tree productivity, it can be concluded that ‘Barkhatnaya’, ‘Inese’, ‘Shater’, ‘Tatyana’, and ‘Vanier’ cultivars are the best for cultivation in the climate of Central Europe. ‘Chuk’, ‘Dofi Sandra’, ‘Early Golden’, ‘Ewierch Rannyj’, ‘Gek’, ‘General’, ‘Kometa’, ‘Kometa Late’, ‘Maschenka’, ‘Naidyona’, ‘Tsernushka’, and ‘Yevraziya’ trees were also very productive. The ‘Barkhatnaya’ and ‘Tatyana’ cultivars turned out to be very susceptible to sharka, and recommending them for commercial cultivation is very risky. Except for pentaploid ‘Herkules’ and aneuploid (nearly hexaploid) ‘Yevraziya’ cultivars, the remaining Japanese plum cultivars turned out to be diploids.

## Figures and Tables

**Table 1:** Health status of 36 *P. salicina* cultivars that were grafted on ‘Wangenheim Prune’ seedlings.

Cultivar.	Frost Damage to the Trees *	Dead Trees	Symptoms of *Plum pox Virus*	
			On the Leaves **	On the Fruits ***
Angeleno	9	0	0	0
Barkhatnaya	9	3	3	3
Black Amber	7	1	0	0
Black Diamond	9	1	0	0
Blue Gigant	7	0	0	0
Chuk	7	0	0	0
Desertnaya Rannaya	9	1	0	0
Dofi Sandra	7	1	3	0
Early Golden	7	2	2	2
Ewierch Rannyj	9	1	0	0
Friar	7	1	0	0
Formosa	7	0	0	0
Gauota	7	1	0	0
Gek	9	0	0	0
General	9	0	0	0
Herkules	9	1	1	1
Inese	9	0	0	0
Kometa	9	2	0	0
Kometa Late	9	0	0	0
Maschenka	9	1	0	0
Naidyona	9	0	0	0
Obilnaya	7	0	0	0
Oishi Wase	9	1	0	0
Ozark Premier	9	1	0	0
Puteshestvennitsa	9	0	0	0
Santa Rosa	9	0	0	0
Shater	7	0	0	0
Shiro	9	0	0	0
Skoroplodnaya	9	1	1	0
Slivovidnaya	9	1	0	0
Superior	7	2	0	0
Tatyana	9	3	3	3
Tsernushka	9	0	0	0
Tsernushka Rannaya	9	0	0	0
Vanier	9	1	0	0
Yevraziya	9	0	0	0

* Frost damage to trees–assessed on a 1–9-point rating scale: 1–withering or withered trees; 3–trees with extensive damage, individual limbs withering, bark on the trunks and limbs heavily cracked and flaking in large patches, and symptoms of the leaves becoming smaller, yellowing and falling off; 5–trees with clear symptoms of damage, withering annual shoots, large spots of discoloured bark, cracked bark peeling and falling off in small patches, symptoms of the leaves becoming smaller, yellowing and falling off; 7–trees with minor injuries, a few small discolorations of the bark on the shoots and signs of the leaves yellowing; 9–no symptoms; ** Symptoms of *Plum pox virus* on the leaves assessed on a 1–3-point rating scale: 0–no symptoms; 1–symptoms on one branch; 2–symptoms on several skeleton branches; 3–symptoms manifested overall; *** Symptoms of *Plum pox virus* on the fruits assessed on a 1–3-point rating scale: 0–no symptoms, 1–superficial symptoms without deterioration of the fruit quality; 2–to 10% of the fruit with sharka symptoms, fruit abscission and low quality for consumption; 3–over 10% of fruits with pathological changes.

**Table 2:** Pomological characteristics and productivity of 36 *P. salicina* cultivars (average, 2015–2020).

Cultivar	Growth Vigour *	Crown Habit **	Date of Flowering			Duration of Flowering (days)	Beginning of Fruiting (Year after Planting)	Productivity ***	Time of Harvest
			Start	Full	End				
Angeleno	7	7	12.04	14.04	22.04	8.5	2	4.2	b. X
Barkhatnaya	3	7	05.04	07.04	15.04	9.3	1	9	b. VIII
Black Amber	5	3	12.04	14.04	23.04	11.7	1	4.6	b. IX
Black Diamond	5	7	11.04	13.04	20.04	12.3	2	3.8	II dec. VIII
Blue Gigant	3	3	12.04	13.04	23.04	11.0	3	3.5	II dec. VIII
Chuk	5	7	04.04	06.04	12.04	10.0	2	8.5	e. VII
Desertnaya Rannaya	7	7	06.04	08.04	19.04	10.7	1	5	e. VII
Dofi Sandra	7	5	06.04	08.04	19.04	10.0	2	8	e. VII
Early Golden	5	7	11.04	13.04	23.04	10.8	1	8	b. VIII
Ewierch Rannyj	7	7	10.04	12.04	19.04	9.0	2	7	b. VII
Friar	5	7	12.04	14.04	23.04	11.7	2	4	e. IX
Formosa	7	7	08.04	11.04	19.04	9.0	2	2.3	b. VIII
Gauota	5	3	11.04	13.04	21.04	10.0	2	5	b. VII
Gek	5	7	13.04	15.04	25.04	9.7	1	7.5	e. VII
General	7	7	06.04	09.04	21.04	14.3	1	7	e. VII
Herkules	9	3	12.04	14.04	23.04	8.7	1	3.3	II dec. VIII
Inese	7	7	11.04	13.04	19.04	9.3	1	9	b. VIII
Kometa	5	7	11.04	13.04	21.04	10.3	1	7.5	e. VII
Kometa Late	5	7	10.04	12.04	24.04	13.0	1	7.5	b. VIII
Maschenka	7	7	10.04	12.04	22.04	11.,3	1	7	II dec. VII
Naidyona	5	5	10.04	12.04	19.04	8.0	2	7	e. VII
Obilnaya	5	7	08.04	10.04	21.04	10.7	1	5	e. VII
Oishi Wase	5	7	08.04	10.04	16.04	10.0	1	4.3	b. VII
Ozark Premier	5	7	08.04	10.04	16.04	9.0	1	6	b. VIII
Puteshestvennitsa	7	7	10.04	12.04	21.04	11.3	1	6.5	II dec. VII
Santa Rosa	7	7	09.04	11.04	22.04	11.7	1	6	II dec. VII
Shater	3	7	08.04	10.04	21.04	11.3	1	9	II dec. VII
Shiro	7	7	11.04	13.04	25.04	12.7	1	6	b. VIII
Skoroplodnaya	5	5	11.04	13.04	21.04	8.0	2	5	II dec. VII
Slivovidnaya	7	7	11.04	13.04	22.04	11.3	2	6	e. VII
Superior	5	7	11.04	13.04	19.04	9.3	1	6	II dec. VIII
Tatyana	3	7	04.04	07.04	16.04	12.0	1	9	b. VIII
Tsernushka	7	7	06.04	08.04	19.04	11.6	1	8	b. VII
Tsernushka Rannaya	7	7	05.04	07.04	14.04	9.7	2	7.9	b. VII
Vanier	5	5	11.04	13.04	21.04	9.5	1	9	II dec. VIII
Yevraziya	7	7	13.04	15.04	25.04	9.3	3	7	e. VII

* Tree vigour–assessed on a 1–9-point rating scale: 1–very weak; 3–weak; 5–medium; 7–strong; 9–very strong; ** Crown habit: 1–columnar; 3–upright; 5–conical; 7–spreading; 9–droopy; *** Productivity: 0–no fruiting; 1–very weak; 3–weak; 5–medium; 7–abundant; 9–very abundant.

**Table 3:** Fruit characteristics of 36 *P. salicina* cultivars (average, 2015–2020).

Cultivar	Mean Fruit Weight [g]	Shape *	Skin Colour **	Flesh Colour ***	Soluble Solids [%]	Stone Separating from Flesh ****
Angeleno	57.3 de	1	9	2/6	14.3 c–i	1
Barkhatnaya	27.7 l–p	2	9	2	14.0 d–j	7
Black Amber	76.7 ab	2	11	2	13.9 d–k	1
Black Diamond	72.1 bc	1	9	6	10.6 n–o	1
Blue Gigant	87.7 a	5	11	6	11.7 m–n	1
Chuk	21.0 p	2	8	2	17.8 a	1
Desertnaya Rannaya	42.0 f–j	1	8	2/6	12.9 h–l	1
Dofi Sandra	39.0 f–l	4	10	2	14.8 c–g	1
Early Golden	41.3 f–k	2	4	2	12.4 j–m	3
Ewierch Rannyj	29.8 j–p	2	9	2/6	15.5 b–e	1
Friar	64.1 cd	1	11	2	13.2 g–m	1
Formosa	51.4 ef	5	6	2	13.5 f–m	1
Gauota	46.6 efg	2	6	2	12.4 j–m	5
Gek	28.3 k–p	4	4	2	13.6 f–l	5
General	51.8 ef	5	8	2/6	13.0 g–m	1
Herkules	78.8 ab	2	6	2	12,6 i–m	1
Inese	21.3 p	4	6	2	15.1 c–f	1
Kometa	35.2 g–o	2	6	2	11.8 l–n	1
Kometa Late	36.6 g–m	5	8	2/6	12.1 k–n	3
Maschenka	24.0 n–p	2	6	2/6	13.5 f–m	1
Naidyona	36.1 g–n	2	6	2	13.9 d–k	1
Obilnaya	43.4 f–i	2	9	2/6	13.6 f–l	7
Oishi Wase	49.7 ef	5	6	2	16.0 bc	1
Ozark Premier	73.2 bc	2	8	2	15.7 bcd	5
Puteshestvennitsa	27.9 l–p	4	8	2/6	17.0 ab	1
Santa Rosa	44.1 fgh	5	9	6	15.2 c–f	1
Shater	28.0 l–p	4	10	2	9.7 o	5
Shiro	35.2 g–o	5	4	2	13.0 g–m	1
Skoroplodnaya	26.7 l–p	2	5/6	2	9.6 o	5
Slivovidnaya	23.2 n–p	2	8	2	13.7 e–k	9
Superior	75.8 bc	5	8	2	15.7 bcd	1
Tatyana	32.2 h–p	1	6	2	14.7 c–h	3
Tsernushka	22.5 o–p	2	8	2	15.9 bc	1
Tsernushka Rannaya	25.9 l–p	2	8	2	15.1 c–f	1
Vanier	51.9 ef	2	3	2	14.0 d–j	1
Yevraziya	30.8 i–p	2	10	2	15.2 c–f	5

Means separation within columns by Duncan’s test at significance level *p* = 0.05; the means marked with the same letter do not differ significantly. * Shape: 1–oblate; 2–circular; 3–elliptic; 4–oval; 5–cordate; 6–oblong; 7–ovate; 8–bottle-like; ** Skin colour: 1–greenish white; 2–green; 3–yellowish green; 4–yellow; 5–orange yellow; 6–red; 7–light violet; 8–purplish violet; 9–dark violet; 10–violet blue; 11–dark blue; *** Flesh colour: 1–whitish; 2–green; 3–yellowish green; 4–yellow; 5–orange; 6–red; **** Stone separating from flesh: 1–very week (adherent); 3–week; 5–medium; 7–good; 9–very good (non –adherent).

**Table 4:** Ploidy level evaluation of *P. salicina* cultivars using flow cytometry analysis.

No.	Cultivar	Ploidy Level	No.	Cultivar	Ploidy Level
1	Angeleno	2×	19	Kometa Late	2×
2	Barkhatnaya *	-	20	Maschenka	2×
3	Black Amber	2×	21	Naidyona	2×
4	Black Diamond	2×	22	Obilnaya	2×
5	Blue Gigant	2×	23	Oishi Wase	2×
6	Chuk	2×	24	Ozark Premier	2×
7	Desertnaya Rannaya	2×	25	Puteshestvennitsa	2×
8	Dofi Sandra	2×	26	Santa Rosa	2×
9	Early Golden	2×	27	Shiro	2×
10	Ewierch Rannyj	2×	28	Shater *	-
11	Friar	2×	29	Skoroplodnaya	2×
12	Formosa	2×	30	Slivovidnaya	2×
13	Gauota	2×	31	Superior	2×
14	Gek	2×	32	Tatyana *	-
15	General	2×	33	Tsernushka	2×
16	Herkules	5×	34	Tsernushka Rannaya	2×
17	Inese	2×	35	Vanier	2×
18	Kometa	2×	36	Yevraziya	6× aneuploid

* Cultivars not analyzed for ploidy level because trees were destroyed due to infection by the *Plum pox virus* (PPV) causing sharka disease.

**Table 5:** List and information on plum cultivars studied. Source: [1,8,16,17,18,21,32].

No.	Cultivar	Reported Parentage	Country
1	Angeleno	Selection within a population of seedlings resulted from open pollination	USA
2	Barkhatnaya	unknown	Ukraine
3	Black Amber	Friar × Queen Rosa	USA
4	Black Diamond	Angeleno × open pollination	England
5	Blue Gigant	No date	No date
6	Chuk	*P. salicina* × *P.cerasifera* (Skoroplodnaya × Otlichnitsa)	Russia
7	Desertnaya Rannaya	Wickson × *P.cerasifera* Tavricheskaya	Ukraine
8	Dofi Sandra	Black Gold × Burmosa	Italy
9	Early Golden	A changce seedling of Burbank or Shiro	Canada
10	Ewierch Rannyj	No date	Ukraine
11	Friar	Gaviota × Nubiana	USA
12	Formosa	unknown	USA
13	Gauota	unknown	Russia
14	Gek	Skoroplodnaya × *P.cerasifera* Otlichnitsa	Russia
15	General	Obilnaya × open pollination	Ukraine
16	Herkules	Ontario × Formosa	Sweden
17	Inese	Seedling of breeding number PU-16807	Latvia
18	Kometa	*P. salicina* × *P.cerasifera* (Skoroplodnaya × Pionerka)	Russia
19	Kometa Late	Kubanska Kometa × open pollination	Russia
20	Maschenka	No date	Ukraine
21	Naidyona	*P. salicina* × *P.cerasifera* (Skoroplodnaya × Desertnaya)	Belarus
22	Obilnaya	*P. salicina* × *P.cerasifera* (Berbank × Tavricheskaya)	Ukraine
23	Oishi Wase	No date	No date
24	Ozark Premier	Burbank × Methly	USA
25	Puteshestvennitsa	*P. salicina* × *P.cerasifera* (seedling of cultivar Desertnaya)	Russia
26	Santa Rosa	*P. salicina* × *P.simonii* × *P.americana*	USA
27	Shater	Fibing × open pollination	Russia
28	Shiro	A chance seedling	USA
29	Skoroplodnaya	*P. salicina* × *P.ussuriensis* Ussuriyskaya Krasnaya × Klaymeks	Russia
30	Slivovidnaya	Obilnaya x open pollination	Ukraine
31	Superior	*P. salicina* Burbank × *(P. americana* × *P. simonii)* Kaga	USA
32	Tatyana	No date	Ukraine
33	Tsernushka	No date	Ukraine
34	Tsernushka Rannaya	No date	Ukraine
35	Vanier	Burbank × Wickson	Canada
36	Yevraziya	Lakrescent × open pollination	Russia

**Table 6:** Annual temperatures and precipitation at the Experimental Orchard in Dąbrowice in 2015–2020.

Year	Temperature [ °C]			Precipitation [mm]
	Minimum	Maximum	Mean	Total
2015	−11.4	37.9	9.9	384.0
2016	−17.9	34.2	9.3	503.8
2017	−20.9	37.4	9.0	564.0
2018	−20.6	35.5	9.7	364.2
2019	−11.5	39.0	10.6	359.7
2020	−10.1	35.9	9.3	499.4

## Data Availability

Not applicable.
